# Forging green Horizons: Revealing Catalysts of pro-environmental behavior in emerging market

**DOI:** 10.1016/j.heliyon.2024.e36332

**Published:** 2024-08-20

**Authors:** Jianmin Sun, Muddassar Sarfraz, Youli Xu, Afshan Azam

**Affiliations:** a School of Management, Nanjing University of Posts and Telecommunications, Nanjing, China; b School of Management, Zhejiang Shuren University, Hangzhou, China; c School of Management, Zhejiang Shuren University, Hangzhou, China; d College of Business, AlYamamah University, Riyadh, Kingdom of Saudi Arabia

**Keywords:** Sustainable consumption, Environmental sustainability, Consumer behavior, Affective commitment

## Abstract

In recent years, environmental pollution has started to threaten global economies. The understanding of consumer behavior within the context of sustainable development has become increasingly important to deal with this growing ecological complexity. Urbanization has accelerated in Pakistan, resulting in urban consumers raising more environmental concerns and promoting eco-friendly products. These concerns have demonstrated their commitment to sustainability and pro-environmental behaviors, such as reducing waste materials (e.g., plastics) and pollutants (i.e., smoke, dust, etc.), thus supporting eco-friendly behaviors. Today, Pakistan's urban consumers are well-aware of environmental complexities. As such, environmental knowledge is the driver of consumers' pro-environmental behavior, affective commitment and social capital also compel individuals to acquire ecological knowledge to enhance consumer behavior. This research considers customers' environmental knowledge and affective commitment, both of which actively contribute to pro-environmental activity. It explores the relationship between environmental knowledge, affection commitment, social capital, and environmental behavior in Pakistan. Data was gathered from Pakistan's urban customers and analyzed using Covariance-based Structural Equation Modeling (CB-SEM). The results indicate that affective commitment and social capital have a positive and significant effect on environmental knowledge and behavior. Notably, the relationship between social capital, affective commitment, and environmental behavior is mediated by knowledge of environmental issues. Through its findings, this study fosters an understanding of environmental behavior and explains the sense of responsibility and greater commitment in individuals, which thus leads them toward sustainability.

## 1 Introduction

Over the years, rapid industrialization has affected the natural environment through regressive ecological liabilities [1]. These environmental vulnerabilities (e.g., deforestation, drought, pollution, and resource depletion) have drastically impacted the world's economies due to damage to the landscape, wildlife, and human health [2]. The ecological pressures originating from industrial chemicals have widely affected humans, their functioning, and their well-being. For example, environmental pollution has inflicted a wide range of adverse health effects, from respiratory diseases to heart and neurological disorders [3]. Moreover, climate change has allowed industrial waste to penetrate deeply into humans' lungs and bloodstreams, exacerbating health conditions [4]. These health implications caused by excessive use of natural resources have raised stakeholders' concerns about environmental protection [5] and shifted their ecological behaviors [6] toward sustainable development [7].

Environmental behavior refers to an individual's actions toward the environment [8]. Consumers minimize the extent and range of environmental problems by practicing pro-environmental behavior [9]. As consumer behavior is directly linked to the protection of the environment [10], consumers' concern for the environment has caused them to pay greater attention to the growing issues with the climate [9]. Due to becoming more concerned about the environment, people are today gaining more knowledge about environmental issues. Environmental knowledge is information that helps individuals influence the natural environment in a positive way [11]. According to Amoah and Addoah [12], knowledgeable consumers who are worried about the growing environmental issues adopt well-informed practices and environmentally-friendly behaviors. Verma and Chandra [13] show that environmental knowledge strengthens consumers' evaluation process, which contributes to eco-friendly behavior.

Di Crosta et al. [14] demonstrate that this change in consumer mindset has revolutionized consumer environmental preferences, attitudes, and knowledge. This change has shaped consumer behavior, thus making consumer psychology the central theme, tailoring real-world behaviors [15]. Consumer psychology has been under discussion for several years. In recent years, it has gained attention, reflecting the shifts in the market dynamics, technological advancements, and evolving consumer expectations [16]. As markets are expanding, the variations in consumer behavior have extended globally. Now consumer environmental psychology can be used as a measurement of consumer concerns in this area, adding to environmental behavior research [17].

White et al. [18] state that this shift in consumer psychology leverages consumers' feelings, thus leading them to engage in substantial pro-environmental behavior. Hopkins [19] suggests that irresponsible human behavior is impacting climate change in ways that are more apparent to the consumer, thus influencing consumer emotions. As consumers are directly affected by the adversity of these growing environmental issues, a more profound shift has occurred in consumers' emotions, leading to better environmental behavior. A key term here is affective commitment, which refers to the consumer's emotional involvement with the object Su et al. [20]. In consumer psychology, affective commitment fosters individuals' sense of obligation toward the environment [21]. It enhances the consumer's feelings about the environment, thereby establishing positive environmental behavior [22].

Given this, Fei et al. [23] claim that social capital also guides individuals' behavioral change. Social capital embeds consumers in a relationship that encourages ecological behavior [24]. Consumers are strongly influenced by their social relations, as the need to gain social capital shapes their feelings and behaviors, thus encouraging them to abide by the norms of their social group [25]. It makes consumers opt for ecological practices, thus reinforcing the development of positive environmental behavior [26].

Various scholars have acknowledged the effect of environmental behavior in terms of consumer psychology [27]. However, to our knowledge, the aspect of emotional affiliation with the environment is yet to be explored fully. The emotional appeal factor in consumer psychology presents interesting findings on ecological behavior. In previous studies, affective commitment has increasingly gained consumers' attention, thus provoking environmentally-friendly behaviors [28]. However, as Clayton et al. [29] show, limited studies have examined the association between consumer affective commitment and environmental behavior. Therefore, this research gap makes it necessary to understand the consumer psychology mechanism (i.e., affective commitment) with consumer environmental-friendly behavior in light of the sustainable development literature.

This study presents a well-grounded theoretical model that enriches the literature on consumer psychology and behavioral sciences in the context of sustainable development. The study objective is to bridge the gap by identifying the measures that have recently gained attention in consumer behavioral psychology. Therefore, the current study presents details on the effective role of the psychological factors that not only enhance consumer behavior but also gain sustainable development. In light of the Theory of Reasoned Action (TRA) and Theory of Planned Behavior (TPB), this study develops a conceptual framework that investigates the relationship between affective commitment and social capital with ecological behavior against the background of sustainable development. In line with this, it also examines the mediating role of knowledge of environmental issues’ nexus to affective commitment, social capital, and pro-environmental behavior.

Understanding these concepts has become crucial for promoting consumer-sustainable choices and actions. Although previous literature has deeply investigated consumer environmental behavior in different contexts [30,31], to our knowledge, this study is the first to investigate environmental behavior alongside the essential elements of consumer psychology under one model. In doing so, this study unifies the most recent knowledge on the fundamental variables that have been previously discussed in different scholarly works. Using the Theory of Reasoned Action (TRA) and Theory of Planned Behavior (TPB), this work offers a valuable addition to the literature by investigating the mediating role of knowledge of environmental issues, which is the prime significance of our study. Overall, this study considers the topic from the level of environmental knowledge and affective commitment, which are active contributors to achieving environmental behavior. As a result, the study outcome is of significance as it enables practitioners and researchers to focus on the growing topic of consumer psychology. Further, it allows marketers and policymakers to understand the role of consumer behavior with unique interventions. Most importantly, it works as a guide to help improve consumers’ environmental psychology and behavior.

## 2 Literature review and theoretical development

The study background is explained in this paper using the Theory of Reasoned Action (TRA) and the Theory of Planned Behavior (TPB). Both theories are the most widely used frameworks that ground the systematic information on human behavior. The TRA argues that individuals' specific behavior is based on their beliefs and attitudes [32]. Meanwhile, the TPB alludes to the individual intention to perform a behavior [33]. In the consumer psychology literature, these perceived behaviors control (i.e., TRA and TPB) predict consumer environmental behavior. They influence one's ecological consciousness and actions [34].

The TRA is closely related to the TPB and provides insight into individual behavior. The TPB, as an extension of the TRA, offers unique contributions to understanding this phenomenon. The TRA indicates that an individual showcases a positive attitude if they perceive social pressure [35]. The TPB, by considering perceived behavioral control, alludes to the individual perception to perform the behavior. This study uses both of these theories to study environmental behavior. The choice between the TRA and TPB may depend on the nature of the behavior.

The TPB is a predictor of additional determinants of behavior [36] and offers a more comprehensive understanding of environmental behavior in terms of knowledge of environmental issues, etc. [37]. It works as a behavioral motivation that can make individuals work toward nature's protection [38]. In the context of environmental behavior, the TPB involves a complex decision-making process influenced by affective commitment, social capital, and knowledge about environmental issues. The TPB provides additional insight into behavior changes by validating them in the context of consumer environmental psychology [39].

Together, the TRA and TPB are dynamic models that help us understand behavioral change. They complement human actions, especially in consumer psychology [40]. The TRA and TPB models can also assist organizations to predict behavior based on the favorable and unfavorable feelings of the individual (i.e., affective commitment) and social influence (social capital) [41]. These consumer behavioral considerations (i.e., TPB) allow firms to capitalize on consumer psychological factors (e.g., affective commitment) [42]. Affective commitment and social capital elevate consumer environmental behavior [43,44]. These factors enhance the consumers’ cognitive and environmental knowledge [45], thus making them follow a particular course of action.

Using these theories, this study explores emotional and social factors, thus gaining insights into environmental behavior. Significantly, studying these elements by applying these theories can help organizations to control and regulate consumer perception in addition to predicting their behaviors. The theories provide comprehensive and systematic models that conceptualize the factors that elevate environmental behavior. These theories that form the basis of environmental behavior can be a valuable guide to future researchers. By adopting these theories, this study explores the core drivers of consumer psychology, thereby encouraging future scholars to expand their research in this area.

### 2.1 Affective commitment and environmental behavior

Continuous changes in climatic conditions have caused consumers to establish strong bonds with the environment [21]. Han and Hyun [46] show that the growing environmental vulnerabilities are the most fundamental element diverting consumer behavior toward environmental protection. Affective commitment plays a significant role in this [47]. Affective commitment refers to one's tendency to develop an emotional connection with the brand or organization [20]. The affective commitment allows the individual to sense the perceived value of the object. In consumer psychology, it enables individuals to improve their actions, in this case, contributing to environmental welfare [48].

Affective commitment excites the individual, thus raising their sense of encouragement. It reinforces positive environmental behavior. Taufique study [22] shows that the emotional connection enhances the customer's perception of the environment, thereby stimulating ecological behavior. It makes the consumer realize the worth of protecting a healthy environment. Furthermore, it encourages customers to opt for environmentally-friendly products. As such, Sun et al. [44] state that a consumer's emotional commitment inspires the consumer to buy eco-products, thus promoting positive consumer behavior. Hence, in line with the TRA and TPB, the following hypothesis is proposed.

H1 Affective Commitment has a positive and significant impact on Environmental Behavior.

### 2.2 Affective commitment and knowledge of environmental issues

In recent years, the rate of climatic issues destroying the natural landscape has led consumers to gain extra knowledge regarding the environment [49]. Environmental knowledge, a significant construct providing relevant information about the environment [50], encourages the individual to act responsibly by realizing the need for environmental protection. Positive emotional appeal is a dominant method to enhance individuals' knowledge regarding environmental issues [45]. The environmental emotion makes consumers realize their obligation toward the environment [22]. This sense of belonging with nature diverts the individuals' focus toward understanding climatic issues, norms, and concepts [51]. Han [52] shows that consumers' affective commitment elevates their connectedness to the environment, thus raising their demand for environmental knowledge.

Consumer needs, motivation, and affection significantly accelerate their environmental knowledge. The affective commitment makes the consumer realize the effect of pollution and other environmental issues and the need for reduction. This high emotional value encourages them to gain information concerning the progressing environmental issues [53]. This knowledge then intensifies the consumer's emotional bond with nature, and businesses may adopt strategies to appeal to customers' feelings in this regard [54]. Consequently, based on the literature gathered, the following hypothesis is proposed.

H2 Affective Commitment has a positive and significant impact on Knowledge of Environmental Issues.

### 2.3 Social capital and environmental behavior

Consumer behavior is the product of social interactions, relationships, and obligations [8]. Social capital refers to individual ties, relationships, and engagement [55]. Wan and Du [56] show that social networks shapes a consumer's identity and knowledge, thus potentially leading to sustainable behavioral change. As per the TRA, social capital is a fundamental tool for behavior changes [35]. According to Xu et al. [57], it is a vital construct that guides individual behavior and actions. The need for social capital makes the consumers feel the pressure of socially acceptable behaviors. This significantly raises consumer care for the environment, thus cultivating pro-environmental behavior [58]. A consumer's sense of obligation toward the environment then contributes to sustainable behavior. As such, Marbuah [59] states that social capital strengthens individuals' willingness to contribute to environmental protection by exhibiting positive ecological behavior.

In particular, social capital can be a driver for strengthening environmental governance and promoting the participation of individuals [60]. It inspires the consumer to make environmentally-friendly choices [23]. A consumer with higher social capital actively participates in ecological activities, thus exhibiting pro-environmental behavior [43]. Therefore, based on the TRA and TPB, the following hypothesis is proposed.

H3 Social Capital has a positive and significant impact on Environmental Behavior.

### 2.4 Social capital and knowledge of environmental issues

Social capital plays an instrumental role in how people exchange opinions with others. This information-sharing activity motivates consumers to gain knowledge that heavily influences their sustainability concerns [61]. The information shared by individuals' sheds light on issues, encouraging others to improve their understanding about the environment. Esfandiar et al. [62] state that the need for social capital drives individuals to realize the need for environmental information. Consumers learn a lot from social groups and the effect of social capital fosters the systematic diffusion of ecological information among social groups, families, and communities [63]. Notably, the force of social capital can shift a consumer's attention toward the increasing environmental issues [64], providing the consumer with essential knowledge that impacts the global communities.

Relevant information on issues that need immediate attention can act as social capital for individuals. As such, social capital works as a fundamental mechanism that elevates the spreading of environmental concerns, issues, and problems that may otherwise be inaccessible to individuals (e.g., consumers) [65]. Wong et al. [66] explain that social capital fosters consumer cognition, thus making them work toward environment conservation. A fundamental factor in environmental research is the individual concern for environmental protection [12]. Social capital promotes social cohesion in a way that makes the consumer focus on environmental degradation. It shows how consumers can react to ecological problems by acquiring sustainable knowledge [56]. Altogether, the prior literature indicates that social capital is a valuable means by which awareness regarding the growing environmental challenges is spread. Hence, based on this view of the TRA and TPB, the following hypothesis is proposed.

H4 Social Capital has a positive and significant impact on Knowledge of Environmental Issues.

### 2.5 Knowledge of environmental issues and environmental behavior

Today, concern about environmental decline has become a relevant topic among global consumers [2]. Knowledge about climate issues enhances individuals' understanding of the global ecosystem. It motivates people to understand climatic vulnerabilities and their effects. In general, such consumer knowledge encourages them to adopt environmentally-friendly practices [67]. This is supported by Oliver et al. [68], who show that consumer environmental consciousness motivates them to gain environmental knowledge that ensures eco-friendly behavior. Environmental awareness profoundly compels individuals to perform positive behavioral changes, thus influencing the global atmosphere [69]. Furthermore, it makes consumers perceive protection of nature as their most dominant responsibility [49]. This shows that environmental awareness is pertinent to understanding the concern for the natural environment. Hence, based on this review, the following hypothesis is proposed.

H5 Knowledge of Environmental Issues has a positive and significant impact on Environmental Behavior.

### 2.6 The mediating role of knowledge about environmental issues

Many consumers want to achieve a constructive ecological civilization and have linked their feelings to nature's security. Environmental behavior is a set of ecological actions that is the product of consumers' emotions and tendencies [51]. Studies have shown that environmental behavior deepens a consumer's bond with the natural environment. However, behavioral change does not take place on its own. It is generally elevated by knowledge that is the result of one's emotions [70]. Ecological affective commitment guides the consumer to conduct the behavior that is beneficial for the environment. In explaining this notion, Kautish et al. [71] show that this natural affective bond raises consumer desire for environmental knowledge that has a wide influence on ecological behavior [52]. This human affective commitment shapes consumers' actions and awareness [21]. Environment knowledge elicits psychological feelings that prompt consumers to gain information about the natural environment. The emotional tendency toward environmental protection drives these consumers to gain new knowledge that enhances their perception of sustainability and makes them environmentally conscious [72]. Environmental consciousness is not only pertinent to building one's knowledge about environmental issues but also to ecological behavior [73].

Su et al. [74] explain that social capital plays a significant role in elevating consumer awareness, which is critical for shaping environmental behavior. When social interactions are friendly, consumers share knowledge and information among themselves [61]. The information shared in the social network enhances the individuals' beliefs, thus making them develop values that elevate, in this case, their eco-friendly behaviors [63]. This exchange of information among social groups contributes to favorable outcomes for the environment. The social capital encompassing the network and social interactions enables the individual to gain diverse perspectives that foster their understanding of environmental issues [75].

Similarly, if ecological issues threaten the well-being of humans, people will take actions to guard against this. Knowledge about these issues assists them in making informed decisions that lead to behavior change [76]. Individuals who hold a deeper understanding of ecological matters (e.g., pollution, waste production, etc.) are equipped to make sustainable choices in their lives, for example, to conserve energy. This shows that ecological knowledge shapes individual behavior toward meeting environmental standards [56]. Indeed, there is growing evidence that social belongings strengthens awareness of ecological concerns and facilitates a person's pro-environmental actions [67]. Altogether, based on the TRA and TPB concepts, and the prior literature, the following hypotheses are proposed.

H5(a) Knowledge of Environmental Issues mediates the relationship between affective commitment and environmental behavior.

H5(b) Knowledge of Environmental Issues mediates the relationship between social capital and environmental behavior.

**Fig. 1:**
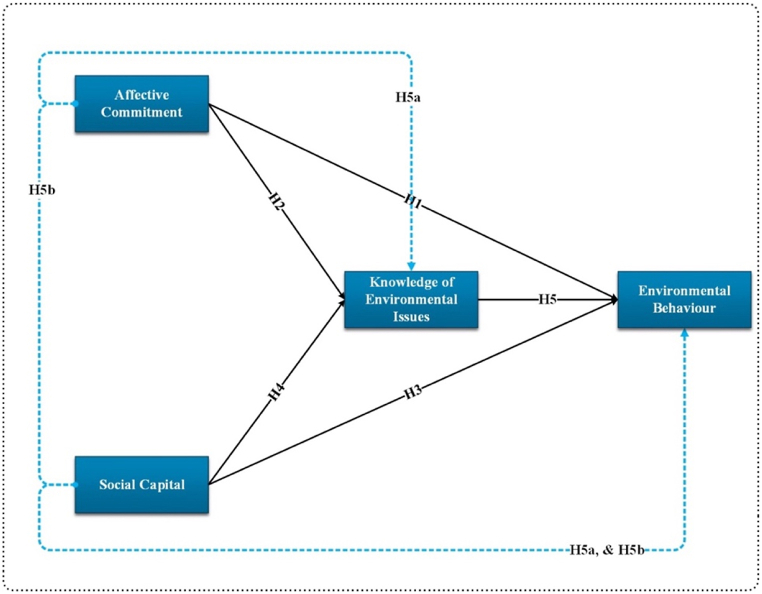
Conceptual framework.

In Fig. 1, the study hypotheses and their relationships are presented along with their underlying relationship.

## 3 Research methodology

The data collection process was designed to ensure accuracy and reliability in capturing relevant information from customers across the major urban cities of Pakistan. The urban population of Pakistan has faced considerable environmental challenges due to growing pollution, waste generation, and resource depletion. As a result, the urban population, which is exposed to massive environmental vulnerabilities, has today realized the need for environmental knowledge and behavior. Environmental behaviors have rapidly evolved in the densely populated city of Pakistan. Urban people have become more environmentally knowledgeable compared to rural people. Therefore, by targeting the urban cities of Pakistan, we aim to tap into the awareness of environmental issues that influence consumer environmental behavior. The study adopted a quantitative and convenient sampling method to collect the data from the urban cities’ customers. A comprehensive survey instrument was developed based on a thorough review of the existing literature and research objectives. A convenient sampling method was employed due to the diverse and expansive nature of the study area. In each city, potential participants were approached in public spaces such as shopping centers, marketplaces, and recreational areas, ensuring representation across different socio-economic backgrounds. Prior to the main data collection, the survey instrument underwent a pilot testing phase with a small sample of participants from each city. Feedback from the pilot study was utilized to refine the survey, ensuring clarity, relevance, and cultural appropriateness.

The teams underwent a comprehensive briefing on the study's goals, ethical considerations, and standardized survey administration procedures. Participants were asked to respond to the structured questionnaire, which covered various aspects of their experiences and preferences. The survey administration took place in a conducive environment to encourage honest and accurate responses. Collected data were entered into a digital database, with a double-entry verification process to minimize errors. Subsequent to data entry, advanced statistical techniques were employed to analyze the data, providing meaningful insights into customer behaviors and preferences.

Participants were provided with a clear explanation of the study's objectives and procedures. Informed consent was obtained from each participant before their inclusion in the study, emphasizing voluntary participation and confidentiality.

In this study, 413 useful questionnaires were collected from the study participants; 188 useful questionnaires were gathered from the male sample (45.5 %) and 225 females (54.5 %). Hence, the study comprises males and females almost equally. The respondents were asked for their age: 51 (12.34 %) were 19–30 years old, 115 (27.84 %) 31–40, and 95 (23 %) 41–50. In terms of marital status, 348 (84.261 %) were married, while 65 (15.7 %) were single (see Table 1).

**Table 1:** Demographic Characteristics.

	Frequency	Percentage
Gender		
Male	188	45.5
Female	225	54.5
***Age***		
19–30	51	12.3
31–40	115	27.8
41–50	95	23
51–60	90	21.8
Over 60	62	15
***Education***		
Intermediate	82	19.9
Bachelor	127	30.8
Master	146	35.4
MPhil/Others	58	14
***Marital Status***		
Single	65	15.7
Married	348	84.3

### 3.1 Study measurement items

The study used the five-point Likert scale to measure the constructs' items. The study adopted the seven-item measurement scale of affect commitment from Fraj-Andrés and Martínez-Salinas [77]. Social capital was measured on the five-item scale while the knowledge of environmental issues scale was measured on an eight-item scale adopted from Castaneda et al. [78]. Fraj-Andrés and Martínez-Salinas's [77] nine-item scale was used for the measurement of environmental behavior. The mediation analysis in our study was conducted using AMOS software. In terms of structural equation modeling, AMOS is widely recognized as having robust capabilities, making it a suitable choice for the current study.

### 3.2 Common method bias

This research also applied the common method bias using Harman's single-factor approach. The variance extracted using one factor is 12.668 %, less than 50 %, indicating no common method bias in this study [79].

## 4 Results

Table 2 shows the reliability and validity analysis of the constructs. The loading value should be > 0.50 to obtain reliable and valid results [80]. Reliability and validity values are based on the loading values [81]. According to the threshold value, the dataset has satisfactory results ranging from 0.719 (for AC_06) to 0.777 (for SC_1). The Cronbach's alpha and CR threshold should be > 0.70 [82]. This dataset shows all values of Cronbach's alpha and CR were greater than the threshold, ranging between 0.924 (for Environmental Behavior) and 0.880 (for Affective commitment), which is above the threshold of 0.7 as suggested by Nunnally and Bernstein [83]. Moreover, the average variance extracted (AVE) value should be > 0.50. As such, the statistics show that there was no reliability and validity issue between constructs.

**Table 2:** Model fit and reliability and validity analysis.

Model fit Indexes				
Fit Index	Cited	Fit criteria	Results	Fit (Yes/No)
X2			410.724	
DF			399	
X2/DF	Kline [84]	1.00–5.00	1.029	Yes
RMSEA	Steiger [85]	<0.08	0.008	Yes
SRMR	Hu and Bentler [86]	<0.08	0.028	Yes
NFI	Bentler and Bonnet [87]	>0.80	0.945	Yes
IFI	Bollen [88]	>0.90	0.998	Yes
TLI	Tucker and Lewis [89]	>0.90	0.998	Yes
CFI	Byrne [90]	>0.90	0.998	Yes

**Alpha, Composite Reliability & Validity Analysis**						
**Construct**	**Items**	**Loading**	**Alpha**	**CR**	**AVE**	**MSV**
1. Affective Commitment	AC_1	0.729***	0.88	0.896	0.551	0.402
	AC_2	0.743***				
	AC_3	0.745***				
	AC_4	0.774***				
	AC_5	0.732***				
	AC_6	0.719***				
	AC_7	0.751***				
2. Social Capital	SC_1	0.777***	0.886	0.886	0.565	0.400
	SC_2	0.734***				
	SC_3	0.769***				
	SC_4	0.745***				
	SC_5	0.749***				
	SC_6	0.737***				
3. Knowledge of Environmental	KEI_1	0.745***	0.911	0.912	0.563	0.407
	KEI_2	0.768***				
	KEI_3	0.714***				
	KEI_4	0.765***				
	KEI_5	0.745***				
	KEI_6	0.737***				
	KEI_7	0.771***				
	KEI_8	0.758***				
4. Environmental Behaviour	EB_1	0.762***	0.924	0.924	0.576	0.407
	EB_2	0.725***				
	EB_3	0.766***				
	EB_4	0.770***				
	EB_5	0.769***				
	EB_6	0.748***				
	EB_7	0.766***				
	EB_8	0.760***				
	EB_9	0.765***				

**Fig. 2:**
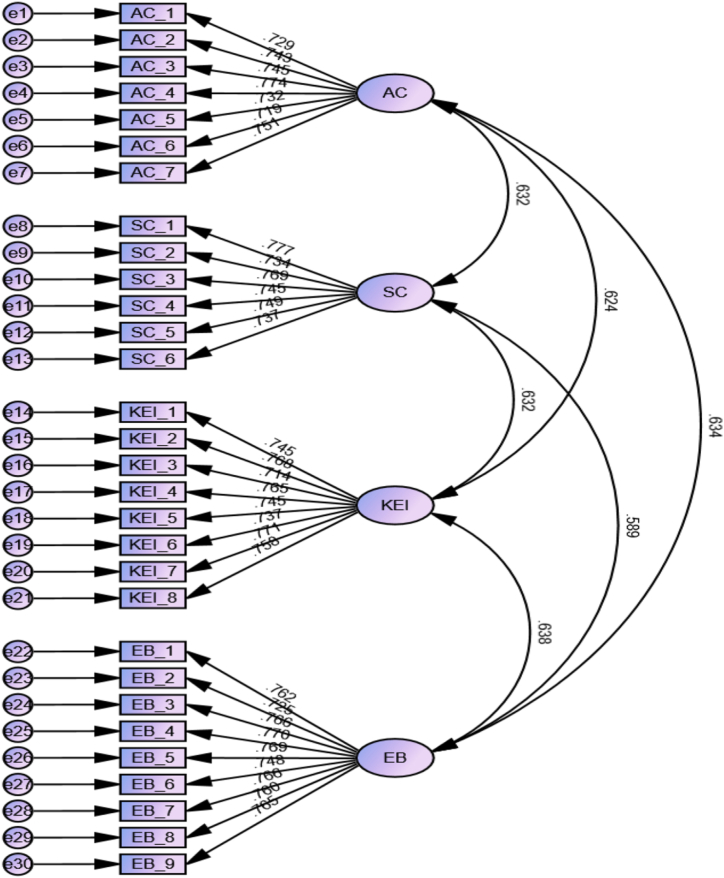
Measurement model graphical representation.

Table 3 shows the analysis of discriminant validity among the constructs. Each construct—namely, affective commitment, social capital, knowledge of environmental, and environmental behavior—is characterized by its mean and standard deviation. The correlations between pairs of constructs are provided, illustrating the strength of relationships between them. The Fornell-Larcker criterion is applied, where discriminant validity is confirmed if the square root of AVE for each construct surpasses its correlations with other constructs. As demonstrated in Table 3, the inter-correlations between the variables range from 0.589 (affective commitment and environmental behavior). The values were lower than the threshold of 0.85. As illustrated in Table 3, each latent construct measurement was completely discriminatory. Fig. 2 is a graphical representation of the measurement model.

**Table 3:** Discriminant validity analysis (Fornell-Larcker).

Constructs	Mean	SD	1	2	3	4
1. Affective commitment	3.611	0.816	**0.742**			
2. Social Capital	3.617	0.855	0.632	**0.752**		
3. Knowledge of Environmental	3.630	0.829	0.624	0.632	**0.750**	
4. Environmental Behaviour	3.634	0.854	0.634	0.589	0.638	**0.759**

Table 4 presents the direct and indirect relationships between variables that measured the significance level by p-value should be less than 0.05. However, hypotheses from H1 to H5 are supported in this study. H1 proposed that affective commitment has a positive and significant impact on environmental behavior. In the study's results, β = 0.335, t = 5.877 should be higher than 1.96, and the p-value is less than 0.05, indicating that H1 is accepted according to the threshold values. In this study, H2 proposed that affective commitment has a positive and significant impact on knowledge of environmental issues. The results indicate that β = 0.386, which shows a positive association, and t = 6.433 is higher than 1.96, while the p-value is less than 0.05, indicating that H2 is accepted. H3 proposed that social capital has a positive and significant impact on environmental behavior. The findings for this hypothesis were that β = 0.178, t-value = 3.296 is higher than 1.96, and the significance level meets the threshold criteria of less than 0.05, so H3 is accepted. Further, H4 proposed social capital has a positive and significant impact on knowledge of environmental issues. The findings indicate that β = 0.422, t = 7.815 higher than 1.96, and the p-value is less than 0.05, meaning that H4 is also supported.

**Table 4:** Hypotheses testing Direct Effect.

Hypothesis	Direct	Std.	Std.	T	P
	Relationships	*Beta*	Error	Values	Values
H1	AC → EB	0.335	0.057	5.877	***
H2	AC → KEI	0.386	0.06	6.433	***
H3	SC → EB	0.178	0.054	3.296	***
H4	SC → KEI	0.422	0.054	7.815	**
H5	KEI → EB	0.335	0.06	5.583	***

*Indicates significant paths: **p < 0.01, ***p < 0.001.

Moreover, H5 proposed that knowledge of environmental issues has a positive and significant impact on environmental behavior. H5 is accepted with β = 0.335, t = 5.583, and a p-value less than 0.05.

As shown in Table 5, all hypothesized mediation effects paths were found to be statistically significant due to having a p-value less than the standard level of 0.05. Therefore, hypotheses H5(a) through H5(b) were all supported. The following sub-sections discuss the results of path analysis concerning the above mediation effect hypotheses: Hypothesis H5(a) shows the relationship between affective commitment and environmental behavior is mediated by knowledge of environmental issues. As shown in Table 5, the result of Bootstrapping indicated that the indirect effect of affective commitment on environmental behavior through knowledge of environmental issues was positive and statistically significant at 0.001 level; β = 0.129, T-value = 3.686, p < 0.001. Therefore, hypothesis H8 was supported.

**Table 5:** Hypotheses testing mediation effect.

Hypothesis	Indirect Relationships	Std. Beta	Std. Error	T- Values	P- Values
H5(a)	AC → KEI → EB	0.129	0.035	3.686	***
H5(b)	SC → KEI → EB	0.141	0.033	4.273	***

*Indicates significant paths: ***p < 0.001.

**Fig. 3:**
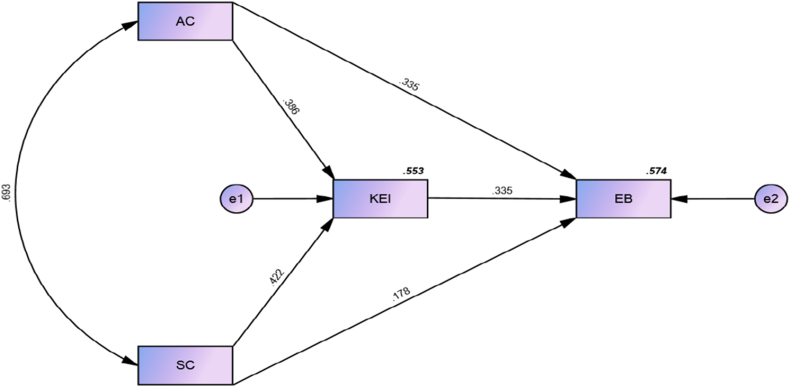
Structural model graphical representation.

Hypothesis H5(b) shows that knowledge of environmental issues mediates the relationship between social capital and environmental behavior. As presented in Table 5, the result of Bootstrapping indicated that the indirect effect of social capital and environmental behavior through knowledge of environmental issues was positive and statistically significant at 0.001 level; β = 0.141, T-value = 4.273, p < 0.001. This suggests that knowledge of environmental issues mediates the relationship between social capital and environmental behavior. Fig. 3 is a graphical representation of the structural model.

Table 6 shows the R^2^ values. Knowledge of environmental issues has an R^2^ value of 0.553 while environmental behavior has an R^2^ value of 0.575.

**Table 6:** R^2^ values.

Latent variables	R^2^
KEI	0.553
EB	0.574

## 5 Discussion

Pakistan's urban consumers are facing pressing environmental issues stemming from urbanization. This ecological footprint has significantly compelled Pakistan's urban consumers to face a wide range of environmental problems, such as air pollution, waste production (e.g., plastics), water contamination, etc. These issues have considerably emerged due to high-centered vehicles, industrial activities, and waste-producing factories, which have widely contributed to poor consumer health. More noticeable climate change effects have led Pakistani urban consumers to more directly experience the decline of the environment, raised the importance they put on environmental behaviors [6]. In the context of sustainable development, global environmental issues are the most critical aspects of behavior change. Sharma et al. [91] use the concept of consumer psychology to explain how consumers' behavior has altered toward the environment. Therefore, given the prominence of the consumer psychological landscape, the current study explored the role of distinctive behavioral factors using the Theory of Reasoned Action (TRA) and Theory of Planned Behavior (TPB). This section discusses the relationship between affective commitment, social capital, and knowledge about environmental issues and environmental behavior against the background of sustainable development concerning the Pakistani's urban consumers.

In recent years, the diminishing natural environment has damaged the worldwide ecosystem [92], elevating the essential need for better environmental behavior. In particular, this challenge of developing environmental behavior has accelerated the need to examine the link between affective commitment and consumer pro-environmental behavior. In consumer psychology, consumers' emotional wants and needs are actively influence behavior. Since a consumer's behavior can be the product of their feelings about the environment, emotional factors are effective drivers of consumer behavior [44]. In this way, a consumer's affective commitment can strengthen their environmentally-friendly behavior [93]. It maximizes the consumer's experience, consumption, and psychology [94]. Kautish and Sharma studies [95] show that emotional attachment enhances consumer behavior toward environmentally-friendly products. This emotional bond causes the consumer to give emotional value to the product, thereby influencing their choice behavior [96].

Nonetheless, with consumer environmental behavior currently gaining considerable attention in academia [97], previous studies have explored some aspects of affective commitment. Our findings support claims in the prior literature that consumer environmental behavior is the product of a consumer's preferences and affection [98] (β = 0.335, p < 0.05). We also found that a consumer's emotional bond increases their individual urge for ecological knowledge (β = 0.386, p < 0.05). Thus, the latest market approach promotes products that satisfy the consumer need for environmental protection [99]. Schneider et al. [21] state that the environment-conscious customer shows a high commitment to the environment. Likewise, Han [52] shows that consumers who feel attached to the environment display a high environmental commitment. Our results confirmed this, highlighting the role of affective commitment in environmental behavior and knowledge of environmental issues (i.e., HI and H2). Under the TRA and TPB model, the research findings shed light on Pakistan's urban consumers' ecological behavior, which has importance in consumer psychology. We found that Pakistani's urban consumers confronting environmental problems have developed a profound affiliation with the environment, influencing them to strive to gain knowledge about ecological issues. Consequently, this awareness of environmental issues has led consumers to recognize the tension between environmental protection and the impact of their consumer behavior [100].

With affective commitment playing a vital role in consumer behavior, our study also found social capital to be an active participant in Pakistani's urban consumer environmental behavior (β = 0.178, p < 0.05). Kautish and Khare study [43] show that social capital influences consumer action toward environmental recovery. Accordingly, Goyal and Goyal [101] claim that an embedded social consciousness significantly alters a consumer's environmental behavior. Our study also found social capital to foster consumer desire for ecological knowledge (β = 0.422, p < 0.05). This is because social capital reinforces consumer choices through sustainable information [102]. Consumers gather new information by listening to other people's opinions, perceptions, and recommendations. This process plays an integral role in fulfilling their need for environmental information [103]. Hence, our results confirm the role of social capital, with H3 and H4 showing that social capital facilitates Pakistani's urban consumer behavior and knowledge about environmental issues, meaning it has become a dominant factor of psychology. In light of TRA and TPB, our study findings indicate that Pakistan's urban consumers rely on social capital to facilitate their ecological behavior and knowledge.

Further, by conducting a deep discussion on environmental behavior, this study confirms that environmental knowledge shapes consumer actions. Stauropoulou et al. [104] state that the increasing ecological concerns inspire individuals to practice sustainability. This high level of consciousness makes consumers realize their responsibility toward the environment [105]. Ecological awareness in turn helps individuals to work toward nature's protection [106]. Ramzan et al. [107] show that consumer environmental knowledge facilitates their eco-friendly behavior. Our findings therefore align with the literature, suggesting that knowledge of environmental issues had a positive and significant impact on environmental behavior (β = 0.335, p < 0.05).

As such, environmental knowledge clearly directs consumers’ attention to ecological behavior. Our findings support Han et al.'s [108] statement that environmental knowledge is a critical factor affecting consumer affective commitment and pro-environmental behavior (β = 0.129, p < 0.001) (i.e., H5a). González-Rodríguez et al. [109] show that to preserve the natural environment, consumers' feeling of connectivity elevates their pro-environmental actions through environmental knowledge. In line with this argument, social capital plays a positive role in environmental knowledge and behavior [74]. Han and Xu [110] state that social capital enables consumers to obtain information that strongly influences their environmental behavior. Hypothesis H5(b) supports this, indicating that knowledge of environmental issues mediates the relationship between social capital and environmental behavior (β = 0.141, p < 0.001).

In light of the TRA and TPB, these research findings are critical to understanding consumers' ecological behavior. The results confirm that consumer behavior is strongly influence by environmental knowledge, affective commitment, and social capital. Therefore, this study highlights the key variables influencing Pakistan's urban consumer pro-environmental behavior. Altogether, the study confirmed that the growing awareness of environmental issues has made Pakistan's urban consumers take ecological actions toward environmental protection. Indeed, the need for pro-environmental knowledge among Pakistani urban consumers is the most needed to navigate the environmental challenges of climate change and industrialization. By taking responsibility for their ecological footprint and making sustainable choices, urban consumers can significantly improve the country's environment and protect the biodiversity, public health, and resources that contribute to sustainability.

### 5.1 Theoretical contributions

The conceptual framework adopted in this study provides a structured and systematic approach to understanding the promotion of sustainable development through consumer behavior. The study findings enhance the depth and breadth of research on this topic, thus guiding scholars, experts, and academic professionals to better deploy the concepts of affective commitment, social capital, knowledge of environmental issues, and environmental behavior in their research. This current study is of great value as it stands out with its comprehensive examination of the unique factors that drive the consumer's pro-environmental behavior. As this study integrates sociocultural factors and local environmental challenges, it offers a fresh perspective on how emerging markets can improve their consumer psychology with sustainable efforts. The current study sheds light on the often-overlooked urban consumer segment of Pakistan, providing valuable insight into different factors driving their pro-environmental behavior. Unlike previous research, which predominately focused on developed nations, this study investigates Pakistan's urban areas. As it widely focuses on Pakistan's urban consumers, it is a valuable guide for other developing countries regarding environmental knowledge and environmental behaviors.

The study findings explored the topic from the level of environmental knowledge and affective commitment, which were found to be active contributors to achieving environmental behavior. Affective commitment significantly engages the customer in sustainable activities. This study is a pioneer as it emphasizes affective commitment in the context of Pakistan's urban consumers. This notion provides a deeper understanding of consumer psychological behavior, which is critical for investigating other emerging markets. Although the previous literature has investigated consumer environmental behavior in different contexts, this study is the first to examine environmental behavior in terms of consumer psychology using the TRA and TPB models. In light of these theories, the current study has presented the most recent findings on these psychological variables. The study, underscoring the importance of environmental knowledge, aims to bridge the literature gap by fostering a culture of responsibility in individuals. Using the Theory of Reasoned Action (TRA) and Theory of Planned Behavior (TPB), this work offers a valuable addition to the literature by investigating the mediating role of knowledge of environmental issues, which is the prime significance of our study. Attitude, as conceptualized in TRA and TPB, encompasses the affective and cognitive evaluations of behavior. This study omits the attitude aspect of these theories. Theoretical models often highlight attitude as ancestors of behavioral intention. Although attitude plays an important role in consumer psychology, it does not always translate into behavioral actions. So, directly concentrating on the behavior, this study provides more actionable insight for adopting factors that have a direct influence on consumer environmental behavior. Moreover, the study excludes perceived behavior control. As the perceived behavior can facilitate or hinder the action, this study does not fully adopt variables from these theories.

Utilizing TRA and TPB, the study findings can help researchers understand how individuals engage in environmentally-friendly behavior while accounting for the role of subjective norms and perceived behavioral control. These motivational factors show how the consumers plan to perform their behavior. However, in the context of this study, these theories are an instrumental guide that forms the basis of one's behavior. Our finding confirms that environmental knowledge, affective commitment, and social capital influence the urban consumer's pro-environmental behavior. By selecting the elements of these theories, the study has developed the cognitive (environmental knowledge), emotional (affective commitment), and subjective norms (social capital) factors to bring positive environmental behavior, which is the prime focus of this study. Under one theoretical framework, this study highlights the role of these factors in the context of the Pakistan urban consumer and TRA and TPB, which is the uniqueness of our study. Indeed, understanding the broader theoretical context of adopted variables, our study selectively integrates variables based on TRA and TPB to highlight the crucial role of environmental issues, social capital, and affective components in pro-environmental behaviors of Pakistani's urban areas.

The integration of these theoretical constructs enhanced our findings. Doing so not only elucidated the predictors of environmental behaviors but also broadened our understanding of consumer psychology in the realm of sustainability. Although past studies have also verified these connections, this study further confirms concepts to assist scholars to realize the value of these notions in terms of environmental sustainability. Emphasizing the critical role of these variables, the current study findings signal the need for greater research on environmentally-friendly behavior. Future scholars should further explore these distinctive measures that reinforce consumer environmental consciousness. There is significant scope for new conceptual frameworks and publications in this field, particularly for the constructs adopted in this research operate in worldwide economies. Overall, this study's findings should encourage academics to consider environmental knowledge concepts in their studies and work toward a universal perspective on environmental awareness as an aspect of consumer behavior.

### 5.2 Managerial implications

This fundamental framework aids in translating the study findings for practical implementation. The study's outcomes should draw stakeholders' attention from a practical point of view, as they offer valuable insights into consumers' emotional connection to nature and how that connection elevates their knowledge of environmental issues and, in turn, pro-environmental behaviors. Therefore, in today's era of increased sustainable possibilities, this study suggests that organizations should adopt effective strategies to evoke consumer environmental behavior and awareness. The findings herein can assist companies to present environmentally-friendly products to consumers, thus raising their affiliation with the environment. Consumers are placing greater demands on companies with regard to ecological practices. The findings outlined in this research can help companies to achieve this goal. Consumers are increasingly showing positive emotions toward more environmentally-friendly offerings, as they desire to behave in an environmentally-friendly manner. As such, the study outcomes should alert management of organizations to the need to respond to consumer-responsible behavior. To achieve that, organizations need to understand consumers' feelings about environmental problems and their effects. Organizations should satisfy consumers' environmental concerns by offering them sustainable choices. Alongside that, they must strengthen their environmental informational network through educational programs to spread environmental knowledge, reinforcing the consumers' environmental behavior.

### 5.3 Policy implications

For businesses to promote sustainable development and practices, affective commitment, social capital, and environmental knowledge play a critical role in ensuring environmental behavior. Investigating these dynamics and interventions better equips firms to promote environmental behaviors. Environmental knowledge is needed in order for consumers to take action to address ecological issues. This ecological value orientation results in enhancing the consumer's emotional bond with the environment and also effectively encourages social interactions that drive knowledge attainment and behavioral changes. As such, the current study contributes to the body of knowledge in this field by identifying opportunities and possibilities to enhance consumer environmental knowledge and behavior.

This study provides valuable knowledge for policymakers, practitioners, managers, and organizations that enables them to study consumer psychology and understand its crucial role in the success of environmental initiatives. In the past decades, interest in the notion of consumer psychology and ecological behavior has risen. This study adds to past research to include further prominent factors that manifest in positive behavior change. The study model enhances the potential pathways for designing policies aimed at improving environmental behavior. Given consumers' desire to enact positive ecological behavior, realization of this sense of ecologically responsible action can be achieved if marketers understand how to signal sustainability in their message. To do this, organizations must identify areas for improvement and design policies that integrate the factors explored herein, thus elevating ecological behavior. The current study's findings suggest that organizations should create coherent and plausible environmental advertising guidelines that inspire people to act sustainably.

## 6 Conclusion

Sustainability demands proper management of the flow of resources in nature. In this regard, environmental psychology is a useful concept for exploring consumers’ attitudes toward sustainability. When consumers upgrade their environmental knowledge, this leads the individual to enact pro-environmental behavior. Growing consumer knowledge about environmental issues and the progressing climatic crisis has increased researchers' interest in investigating the influence of environmental knowledge on consumer ecological behavior. The literature suggests that various types of psychological constructs, such as affective commitment, environmental knowledge, and social capital, play a crucial role in consumers' pro-environmental behavior. Consumers' environmental behavior is thus thought to be deeply influenced by affective commitment. The affective dimension makes individuals aware of environmental issues, thus elevating their eco-friendly behavior. As consumer actions are stimulated by various factors, social capital is also believed to translate consumers' environmental concerns into more responsible behavior.

In the context of Pakistan's urban consumers, the study discusses the concept of consumer psychology and its merits in understanding the environment and behavior in terms of key behavioral theories (i.e., TRA and TPB). The paper offers a systemic review of consumer environmental psychology and behavioral studies that explicate pro-environmental actions. Our study findings show that affective commitment and social capital facilitate consumers' environmental behavior and knowledge. Furthermore, this study confirms the positive mediating role of knowledge of environmental issues' nexus to affective commitment, social capital, and environmental behavior. Significantly, this study presents a broader framework of different factors that can help scholars, businesses, and policymakers understand the role of effective interventions. The study findings also open new avenues for future researchers. It provides valuable insight to organizations, researchers, consumers, environmentalists, and practitioners by bolstering their understanding of the role of environmental psychology in consumers' growing need for sustainability in the modern marketplace.

### 6.1 Future Prospect and study limitations

The adoption of the TRA and TPB model in this study was significant in determining environmental behavior and knowledge; however, it still has a few limitations. Discussing these limitations provides opportunities for future exploration. Firstly, the study results showed that data was gathered from the consumers of Pakistan only. Therefore, to gain generalized results, the study recommends that the geographical focus of the research be broadened to study consumers in different economies (e.g., China, Romania, Bangladesh, etc.). Further, it is recommended that the current study findings should not be only limited to big cities of Pakistan. Future scholars could include other regions and cities to increase the generalizability of the research findings. Furthermore, the study considers the influence of limited variables, investigating the role of only a few psychological factors in consumer environmental behavior. Scholars could add more psychological factors to gain a wider perspective on this topic. As the focus of the study is of great interest worldwide, future researchers could also incorporate different factors (e.g., economic and social background) when considering the impact of environmental issues on environmental behavior.

Similarly, another limitation is that the current paper includes only one mediator (i.e., knowledge of environmental issues). So, to expand the study scope, future scholars could use more variables as mediators, such as environmental consciousness, green satisfaction, trust, etc. They could also incorporate moderators, such as connectedness to nature, eco-innovation, etc., to better understand the topic. Lastly, the study focuses on consumer pro-environmental behavior in general. Future studies should consider the specific types of pro-environmental behavior that are influenced by the driving factors established here. Future research can thus build on the foundations provided in the current study, which offers meaningful insights into the consumer psychological phenomenon. Subsequent studies can further develop this topic in different contexts, thus adding to our understanding of consumer environmental behavior.

## Data availability statement

The data that support the findings of this study are available from the corresponding author upon reasonable request.

## Funding statement

This study was supported by The General Program of the 10.13039/501100012456National Social Science Foundation of China under Grant (23BTJ016).

## Ethical approval

All participants gave their informed consent for inclusion before they participated in the study. All procedures performed were by the ethical standards as laid down in the 1964 Declaration of Helsinki and its later amendments or comparable ethical standard. All the procedures were approved by the ethical committee of School of Management, Zhejiang Shuren University (24-05/71).

## CRediT authorship contribution statement

**Jianmin Sun:** Software, Resources, Funding acquisition, Formal analysis. **Muddassar Sarfraz:** Writing – review & editing, Writing – original draft, Supervision, Project administration, Conceptualization. **Youli Xu:** Writing – review & editing, Resources, Funding acquisition. **Afshan Azam:** Writing – review & editing, Validation.

## Declaration of competing interest

The authors declare the following financial interests/personal relationships which may be considered as potential competing interests:Muddassar Sarfraz is an Associate Editor of Heliyon (Business and Management) section. However, the manuscript was submitted under double-blind peer review process. The authors declare that they have no known competing financial interests or personal relationships that could have appeared to influence the work reported in this paper.
